# Abnormal glucose homeostasis and fasting intolerance in patients with congenital porto-systemic shunts

**DOI:** 10.3389/fendo.2023.1190473

**Published:** 2023-08-18

**Authors:** Mirjam E. van Albada, Pratik Shah, Terry G. J. Derks, Sabine Fuchs, Judith J. M. Jans, Valérie McLin, Hubert P. J. van der Doef

**Affiliations:** ^1^ Department of Pediatric Endocrinology, University Medical Center Groningen, University of Groningen, Groningen, Netherlands; ^2^ Department of Pediatric Endocrinology, The Royal London Childrens Hospital, Barts Health National Health Service (NHS) Trust and William Harvey Research Institute, Queen Mary University London, London, United Kingdom; ^3^ Department of Metabolic Diseases, Beatrix Children’s Hospital, University Medical Center Groningen, University of Groningen, Groningen, Netherlands; ^4^ Department of Metabolic Diseases, Wilhelmina Children’s Hospital, University Medical Center Utrecht, Utrecht, Netherlands; ^5^ Department of Genetics, Section Metabolic Diagnostics, University Medical Center Utrecht, Utrecht, Netherlands; ^6^ Swiss Pediatric Liver Center, Department of Pediatrics, Obstetrics, and Gynecology, University of Geneva, Geneva, Switzerland; ^7^ Department of Pediatric Gastroenterology and Hepatology, University Medical Center Groningen, University of Groningen, Groningen, Netherlands

**Keywords:** hypoglycaemia, hyperinsulinism, congenital porto-systemic shunt, portal circulation, glucose metabolism, insulin, congenital hyperinsulinism

## Abstract

In physiological glucose homeostasis, the liver plays a crucial role in the extraction of glucose from the portal circulation and storage as glycogen to enable release through glycogenolysis upon fasting. In addition, insulin secreted by the pancreas is partly eliminated from the systemic circulation by hepatic first-pass. Therefore, patients with a congenital porto-systemic shunt present a unique combination of (a) postabsorptive hyperinsulinemic hypoglycaemia (HH) because of decreased insulin elimination and (b) fasting (ketotic) hypoglycaemia because of decreased glycogenolysis. Patients with porto-systemic shunts therefore provide important insight into the role of the portal circulation and hepatic function in different phases of glucose homeostasis.

## Introduction

The liver has multiple roles in endocrine physiology, which are increasingly recognized (1). These roles include hormone synthesis (e.g. IGF-1), degradation (e.g. insulin or sex steroids), precursor synthesis (e.g. cholesterol and lipoproteins for glucocorticoid synthesis), and binding protein synthesis. In normal physiology, the liver receives 25% of its afferent blood supply from the hepatic artery, and 75% from the portal vein (2). Necessary endocrine metabolites enter the liver either by the portal vein or by the hepatic artery. In patients with a congenital portosystemic shunt (CPSS), the liver is bypassed to varying degrees, leading to metabolite and hormonal abnormalities, including hypoglycaemia, hyperammonemia, increased blood bile acids, hypothyroidism and hyperandrogenism, among others (3).

Although hypoglycaemia is a relatively common metabolic abnormality in pediatrics, awareness of CPSS as an underlying cause is low, as illustrated by the fact that CPSS was not a differential diagnosis in a recent review of hypoglycaemia (4). Yet, patients with CPSS are at risk for a unique combination of postabsorptive HH and fasting (ketotic) hypoglycaemia.

Therefore, the primary aim of this manuscript is to summarize the current knowledge of hypoglycaemia in CPSS patients, and the secondary aim of this review is to go deeper into glucose metabolism in CPSS, which in turn enhances our understanding of the liver’s role in glucose and insulin physiology.

## Case report

An 8-year-old child had experienced loss of consciousness after a 15-hour fast during an infectious episode at 9 months of age. Despite normal development and physical examination with no organomegaly, a fasting test revealed hypoglycaemia of 2.6 mmol/L (47 mg/dl) and ketones of 2.2 mmol/L. Glucose levels rose to 11.8 mmol/L (212 mg/dl) upon feeding, leading to suspicion of glycogen storage disease type 0, which was ruled out through genetic testing. An oral glucose tolerance test was performed to investigate the response to feeding and test gluconeogenic and mitochondrial function, revealing hypoglycaemia of 1.9 mmol/L (34 mg/dl) after 120 minutes with a peak glucose of 10.7 mmol/L (193 mg/dl). Continuous glucose sensing showed regular nocturnal hypoglycaemia, and treatment with twice daily corn starch in a gradually increased amount improved wellbeing. Metabolic analyses showed mildly increased ammonia (<100 umol/l) and purines, but genetic investigation was non-contributory. Untargeted metabolomics in a research setting revealed increased bile salt concentrations together with hypoglycaemia and hyperammonemia, leading to a diagnosis of CPSS, as was confirmed by ultrasound imaging. Our experience with similar cases has led to considering CPSS in the differential diagnosis of unexplained hypoglycaemia.

## Patients with CPSS and hypoglycaemia

The first case of HH in CPSS was reported in 1984 by Gouin and Duprey (5, 6). They described a 63-year-old woman with recurrent malaise due to hypoglycaemic episodes, which was ultimately attributed to a portosystemic shunt thought to be congenital in origin. The patient’s glycoregulation showed impaired glucose tolerance, followed by post-stimulatory hypoglycaemia with persistent hyperinsulinemia. Since then, more than 10 cases have been reported by various authors (3, 7–13). In many of these cases, hypoglycaemia was the presenting symptom. However, it is currently unknown how many patients with CPSS suffer from HH, as screening for hypoglycaemia is not standard in CPSS patients. For instance, Sokollik et al. (14) did not report hypoglycaemic episodes in their case series of 22 patients, while Baiges et al. (15) did not find any abnormalities in glucose homeostasis in a series of 66 adults with extra-hepatic CPSS. Similarly, a small series of infants with intra-hepatic CPSS did not mention HH. However, Xu et al. presented a neonatal cohort of 16 patients with CPSS, 40% of whom presented with hypoglycaemia as a clinical symptom (9).

## Glucose homeostasis

Ruderman (16) divided glucose homeostasis into five phases starting from the moment of carbohydrate ingestion: absorptive, post-absorptive, and early, intermediate, and prolonged starvation. The first two phases are essential to understanding glucose metabolism in the setting of CPSS.

In the absorptive phase, glucose and insulin are the most crucial components. Digestive tract glucosidases process ingested carbohydrates. The resulting monosaccharides are transported to the liver via the portal circulation, where they enter hepatocytes through insulin-mediated transport. Glycogen synthase initially converts them into glycogen, and excessive carbohydrate consumption leads to lipogenesis. Under normal conditions, one-third of the glucose in the portal vein is taken up by the liver, and the rest of it is delivered to the rest of the body through arterial blood (17).

Insulin levels rise in response to elevated blood glucose and regulate hepatic glucose uptake. In parallel, pancreatic beta-cells act as glucose sensors, linking changes in arterial blood glucose concentration to the rate of insulin secretion. Pre-proinsulin processes to its mature form, generating c-peptide. Typically, insulin production occurs in less than two hours. Roughly 75 to 95% of total insulin is stored within beta-cells at some distance from the cell membrane, while a smaller part of the insulin is packed in granules that are ready to go. This efficient organization likely underlies the biphasic nature of glucose-evoked insulin secretion that is seen *in vitro*, with a rapid first phase that lasts up to 10 min followed by second phase (18).

The human pancreas secretes approximately 30 units of insulin per day into the portal circulation. Insulin that enters the hepatic sinusoid through the portal circulation accesses hepatocytes through the fenestrated sinusoidal endothelium. It binds to the insulin receptor and, after internalization, is degraded by insulin-degrading enzymes. Normal hepatic insulin clearance is around 50-80% of daily production (19). Peripheral insulin concentration increases within 8-10 minutes after food ingestion, peaks at 30-45 minutes, and rapidly declines to baseline values by 90-120 minutes postprandially (19, 20). Insulin’s primary action is to stimulate glucose uptake and promote glycogen synthesis in the liver and muscles.

When it was demonstrated that oral glucose intake results in a greater and longer-lasting rise in plasma insulin compared to an intravenous administration of the same dose, scientists hypothesized that there must be a gastro-intestinal factor triggered by alimentary glucose. This was later termed the incretin effect (21), with the two most important incretins being Glucagon-like peptide 1 (GLP-1) and GIP, both of which independently contribute to insulin release.

GLP-1 is produced as proglucagon and is primarily generated in the entero-endocrine L cells that are found in the intestine, especially in the distal ileum. The production of GLP-1 is stimulated by the consumption of various nutrients, particularly glucose and fat, as well as bile acids. The GLP-1 receptor is found in numerous organs and stimulates insulin secretion in a glucose-dependent manner. However, GLP-1 is rapidly degraded *in vivo* by dipeptidyl peptidase-4 (DPP-4), an enzyme that is highly expressed in the liver (22). DPP-4 is a crucial determinant in regulating the biological activity of incretins. Thus, liver disease or bypass may impact fasting glucose regulation both through diminished GLP-1 degradation and altered bile acid signaling given the partial interruption in the enterohepatic circulation that occurs in CPSS (23).

In the normal post-absorptive phase, insulin action is suppressed when circulating glucose levels decrease, and the body relies on endogenous glucose production (EGP). EGP first utilizes glycogenolysis (second phase), followed by gluconeogenesis in early starvation (third phase), fatty acid oxidation in intermediate starvation (fourth phase), and ketogenesis and ketolysis after prolonged starvation (fifth phase). A large portion of this endogenously produced glucose is used to provide energy for the brain. The ratio of brain weight to body weight is highest in young children (24), and studies have shown that 3-4 year old children have a glucose appearance rate that is approximately 3 times higher (6-7 mg/kg/min) than in adults (25). As a result, liver glycogen stores are depleted more quickly in children, which can result in the formation of ketone bodies after a few hours of fasting (26), indicating intermediate starvation after a brief period.

Glucagon plays a critical role in maintaining glucose homeostasis in the post-absorptive phase (27). Its primary secretory stimulus is a low plasma glucose concentration, although other variables such as the stress related to trauma or hypoxia may act as triggers (28). Pancreatic glucagon secretion leads to a kinase-mediated release of glucose from stored glycogen. Proglucagon processing leads to the production of GLP-1 and -2 in intestinal L-cells, as well as the production of glucagon in pancreatic alpha cells (29). Glucagon is transported through the portal circulation to the liver, where it exerts its effects via a specific extracellular receptor.

## Glucose metabolism in CPSS

In CPSS patients, hepatic bypass can cause a rapid spike in plasma glucose levels due to the postprandial entry of glucose into the systemic circulation. This effect has been observed in OGTTs and fasting tests, where patient glucose levels spiked significantly (3). This spike in glucose levels is followed by hypoglycaemia, occurring 90-120 minutes after carbohydrate-rich meals that are high in fast-acting carbohydrates and low in other macro-nutrients. The increased insulin levels due to hepatic bypass are incompletely degraded, leading to this hypoglycaemia. It is possible that a decrease in GLP-1 breakdown might help mitigate this effect. This HH might be considered one of the metabolic hallmarks of CPSS.

Additionally, smaller children may be more vulnerable to fasting-induced hypoglycaemia with ketone production, as hepatic glycogenesis may be less efficient in this population. Possibly, altered glucagon metabolism also contributes to a different fasting ability.

It is important to note that the clinical picture of CPSS can be diverse and may vary depending on the quantitative portal flow and the presence of additional metabolites and pathways affected by portocaval shunting, such as fatty acid oxidation and amino acid metabolism, which are beyond the scope of this review. CPSS patients can present a wide spectrum of relatively nonspecific symptoms, signs and complications. These may occur throughout life and include unexplained neurocognitive dysfunction and behavioral problems, neonatal cholestasis, and galactosemia. Some of these symptoms and signs are mimicking several rare inherited metabolic disorders, in which a substrate shunt is caused by a transporter or enzyme deficiency at a cellular level. **Table 1** summarizes the key-features of four inherited metabolic disorders in very different metabolic pathways, that display interesting similarities and differences with CPSS. Both in these metabolic patients and CPSS patients, the metabolic profile depends highly on the timing of blood sampling with respect to the last meal.

**Table 1:** Examples of metabolic diseases with molecular glucose shunting.

Name	Glucose transporter 2 deficiency	Hepatic glycogen synthase deficiency	Glutamate dehydrogenase superactivity	Transmembrane protein 70 deficiency
**Disease abbreviation**	FBS (Fanconi-Bickel Syndrome)	GSD-0a	GLUD1	NME (Neonatal Mitochondrial Encephalopathy)
**Gene**	*SLC2A2*	*GYS2*	*GLUD1*	*TMEM70*
**OMIM**	# 227810	# 240600	# 606762	# 614052
**Inheritance**	Autosomal recessive	Autosomal recessive	Autosomal dominant	Autosomal recessive
**Hepatocyte location**	Cell membrane	Cytosol	Mitochondrial	Mitochondrial
**Clinical symptoms**	Hepatomegaly n -↑↑↑ Renal tubulopathy, generalized ± Short stature n-+++ Nefromegaly Reduced bone density Rickets Thin limbs	Hepatomegaly n Hypoglycemia, fasting ± Seizures ±	Epilepsy, generalized ± - ++ Hyperinsulinism ± - ++ Hypoglycemia + - ++ Hypoglycemia, hypoketotic + - ++ Leucine sensitivity causing hypoglycemia ++	Cardiomyopathy, hypertrophic + Encephalopathy + Failure to thrive + Hypotonia, muscular-axial + Ketonuria, pronounced during crisis + Lactic acidosis ± - + Metabolic acidosis ± - + Psychomotor delay ± - + Low birth weight Facial dysmorphic features Ataxia in those who survive Intention tremor in those who survive Leukoencephalopathy
**Biochemical markers**	Plasma glucose: - Fed n-↑ - Fasted n-↓ Plasma galactose ± Plasma uric acid ↑ Serum: - AST/ALT n -↑ - Cholesterol n -↑ - Triglycerides n -↑ - AF n -↑ Urine: - Glucose ↑-↑↑↑ - Calcium n -↑ - Phosphate n -↑ - Amino acids n -↑	Plasma glucose: - Fed n -↑ - Fasted n -↓ Plasma lactate: - Fed n -↑ Plasma ketones: - Fasted n -↑ Urine ketones: - Fasted n -↑	Plasma glucose ↓ Serum free fatty acids ↓ Blood/plasma ammonia - Fasted ↑ Plasma/urine ketones ↓	Plasma amino acids: Ala ↑-↑↑, Cit n -↑, Glu n-↑ Plasma CK n -↑↑ Plasma lactate Glucose ↑-↑↑↑ Blood ammonia n -↑↑ Anion gap ↑ Urine: - 3-methylglutaconic acid ↑-↑↑ - Orotic acid n-↑ - Uric acid n-↑↑

(n, normal; ↑, mildly increased; ↑↑, increased; ↑↑↑, severely increased; ↓, decreased; ±, sometimes present; +, often present; ++, always.

## Diagnosing hypoglycaemia in CPSS

Acute hypoglycaemia is a common pediatric metabolic emergency with a wide range of possible causes. Rare conditions like CPSS and inherited metabolic disorders are not always taken into consideration in the differential diagnosis. Furthermore, the definition of childhood hypoglycaemia is not always clear cut. The normal range for fasting glucose levels is between 3.5 mmol/L and 5.5 mmol/L (63 mg/dL and 100 mg/dL) (30). Generally, for neonates over 48 hours old, infants, and young children, a glucose level below 3.3 mmol/L (60 mg/dL) is considered too low (31). However, it is worth noting that healthy children between the ages of 8 and 15 can occasionally have glucose levels ≤ 3.3 mmol/L or 60 mg/dL in 0.2% of the time on continuous glucose monitoring (CGM), and ≤ 3.9 mmol/L or 70 mg/dL in nearly 2% of the time (32–34). The presence of Whipple’s triad (classical symptoms of hypoglycaemia, symptoms at the time of a low blood glucose concentration, and relief of symptoms after the increase of the glucose level), when present, can be helpful in diagnosing hypoglycaemia in older children (30).

It is essential to monitor patients at risk for hypoglycaemia, especially in pre-school children, as they are vulnerable to the consequences of hypoglycaemia on their developing central nervous system. Patients with congenital hyperinsulinism are known to be extremely susceptible to the effects of hypoglycaemia on the brain because insulin also decreases the production of ketones, the alternative fuel for the brain (35, 36). This is further illustrated by the high prevalence of neurocognitive dysfunction in these patients (37). In patients with CPSS, the symptoms of hypoglycaemia can be difficult to distinguish from symptoms related to minimal hepatic encephalopathy in the presence of elevated plasma ammonia, one of the other metabolic characteristics of patients with CPSS.

Patients at risk for hypoglycaemia can be monitored using frequent finger prick blood glucose testing, oral glucose tolerance test, fasting test, or continuous glucose monitoring (CGM). CGM use is rapidly expanding due to its continuous measurements and relatively uninvasive nature. In patients with diabetes mellitus, CGM is the standard of care. For the early recognition and reduction of HH, CGM is also becoming increasingly established (38). In rare or unexplained diseases with hypoglycaemia, CGM can provide additional information, helping to determine the etiology and to optimize treatment for the individual patient, as recently reported for patients with hepatic glycogen storage disease (39).

**Figure 1:**
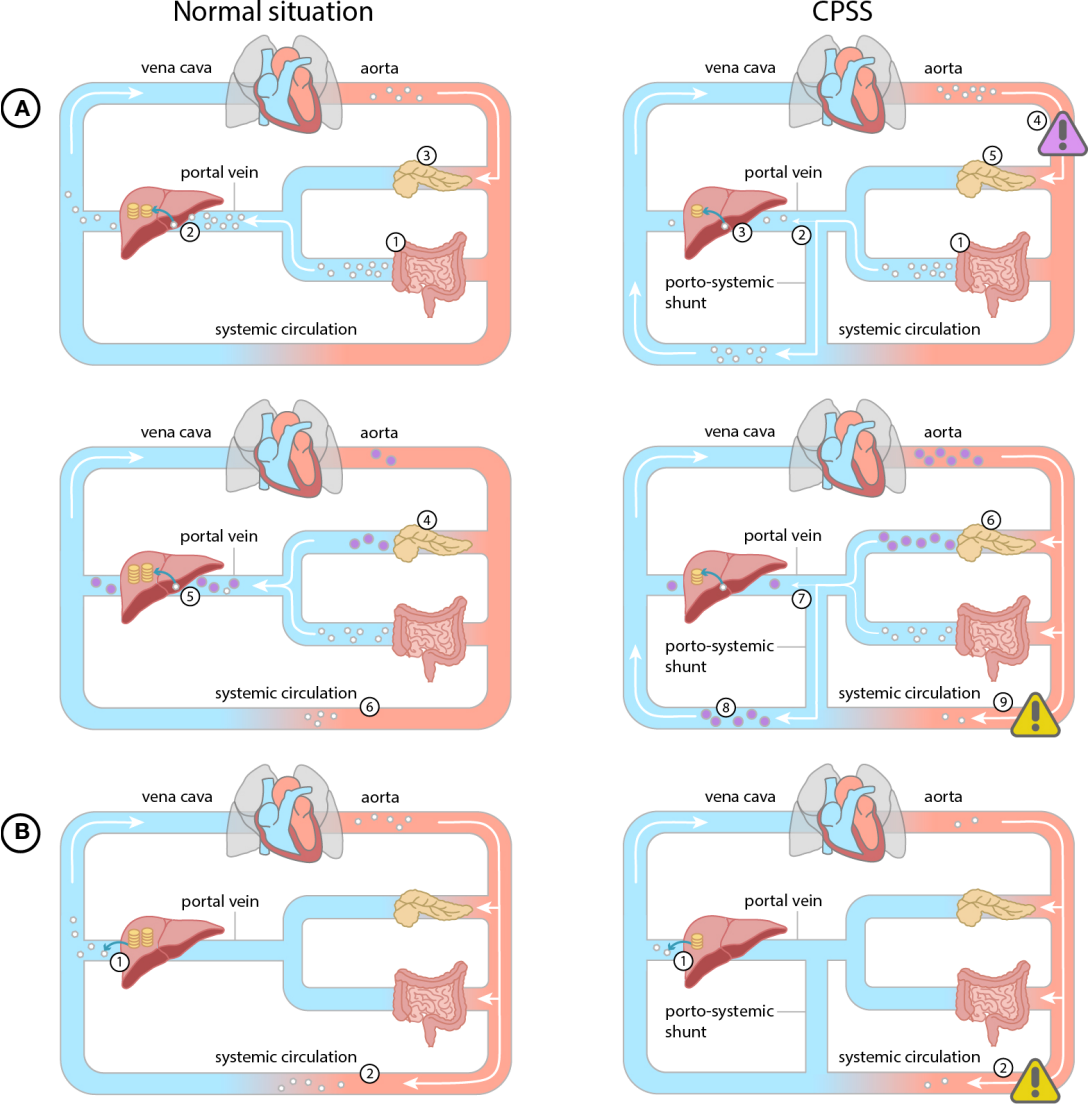
Normal situation (glucose = white dot, insulin = purple dot, glycogen = yellow coin): **(A)** Post prandial. 1) Glucose is absorbed from the intestines into the mesenteric veins. 2) Glucose is converted and stored as glycogen in the liver during the first pass effect. 3) Pancreatic beta cells detect the increased glucose levels. 4) Pancreatic beta cells secrete insulin according to glucose levels. 5) Hepatic insulin metabolism reduces systemic insulin levels. **(B)** During fasting. 1) Stored glycogen is converted to glucose and enters the bloodstream. 2) This mechanism prevents hypoglycemia during fasting. CPSS **(A)** Post prandial glucose. 1) Glucose is absorbed from the intestines into the mesenteric veins. 2) Due to the porto-systemic shunt the glucose partly bypasses the liver, 3) causing less glucose to be stored as glycogen in the liver 4) and a systemic hyperglycemia (purple warning sign). 5) Pancreatic beta cells detect the increased glucose levels. 6) Pancreatic beta cells excrete more insulin. 7) The insulin bypasses the hepatic metabolism directly flowing into the systemic circulation 8) leading to systemic hyperinsulinemia. 9) Hyperinsulinemia results in a late hypoglycemia in end-organs (yellow warning sign). **(B)** During fasting. 1) Due to reduced glycogen storage in the liver, significantly less glycogen can be converted to glucose to enter the bloodstream 2) resulting in a hypoglycemia in end-organs (yellow warning sign).

The oral glucose tolerance test with measurement of glucose and insulin at every time point is most frequently used to establish the diagnosis of hypoglycemia in CPSS (3, 7, 8, 10, 12), with a typical pattern of postprandial hyperglycemia followed by hypoglycemia (**Figure 1**). However, glucose and insulin are only measured every 30 minutes during 2.5-3 hours after glucose ingestion. CGM has the advantage of measuring glucose levels for several days in daily life practice, providing a better understanding of the fluctuations of glucose levels. This ultimately leads to a better understanding of the disease and the optimization of management for each individual patient.

## Treatment of hypoglycaemia in CPSS

Symptomatic CPSS need to be closed either using an endovascular or surgical approach. This has been documented to resolve hypoglycaemia (10–13). Dietary management of hypoglycaemia can be considered in rare cases of contraindication to closure or as a bridge to closure. Two studies have reported on the management of HH related to CPSS (8, 11). The dietary approach involves a diet low in simple carbohydrates and rich in complex carbohydrates, with the prevention of longer fasting. Specifically, the use of galactose-free milk and corn starch is recommended (8). Drug treatment may consist of alpha-glucosidase inhibitors or diazoxide, although only one patient received diazoxide in the study of Weigert et al. (11).

Meals with slow-acting carbohydrates with a low glycemic index may prevent insulin spiking in these patients. Adding ultra-slow acting carbohydrates, such as corn starch or glycosade, to the diet can help prevent extremely low glucose levels and hypoglycaemia after a night of fasting. Corn starch therapy should be initiated with small doses, gradually increased, and carefully monitored, as the enzyme needed for intestinal digestion of cornstarch may not be fully present before 2 years of age. The effects on glucose homeostasis, fasting tolerance, and symptoms (including mood and growth) should be carefully balanced against side effects, such as intestinal gas, bloating, and diarrhea.

Diazoxide is one of the most potent agonists of the K+ ATP channels on the insulin-producing beta-cells of the pancreas. Its activation reduces insulin secretion and theoretically, it could be used to prevent hyperinsulinism and hypoglycaemia in CPSS patients. However, it may further increase the high spikes in glucose, potentially aggravating the fluctuations. There have been some reports with variable effects on postprandial hypoglycaemia. The main side effect is fluid retention, sometimes complicated by hypertrichosis or pulmonary hypertension (PoPH). Given the association between PoPH and CPSS, avoiding diazoxide seems reasonable. Alpha-glucosidase inhibitors are another treatment option, although their effectiveness in CPSS is not well established.

## Conclusions and perspectives

Congenital porto-systemic shunts are vascular anomalies that can affect glucose and insulin metabolism, and are most typically characterized by episodes of HH and variable fasting intolerance. Despite their rarity, they should be considered as a potential cause of hypoglycaemia and are easily diagnosed using Doppler ultrasound. In patients diagnosed with CPSS we advise to screen patients for hypoglycaemia with continuous glucose monitoring or oral glucose tolerance testing with glucose and insulin at every time point. Repeated hypoglycaemia during childhood can have serious neurological and cognitive consequences, particularly if there is not enough ketone production to compensate. Therefore, it is recommended to evaluate glucose and ketone levels after fasting and carbohydrate intake in these patients. Shunt closure is the ultimate solution, but dietary management can also play a significant role in stabilizing glucose levels as a bridge to closure or in rare cases where shunt closure is contraindicated. The intricacies underlying varying individual presentations and tolerance to HH remain to be elucidated, as do the impact of portosystemic bypass on other metabolic pathways involved in buffering the stress of fasting.

## Author contributions

MA, VM and HD drafted and corrected the manuscript PS corrected the manuscript and provided clinical input TD contributed the table and made additions to the manuscript from the metabolic perspective SF provided clinical details from the case and corrected the manuscript JJ performed and interpreted the metabolomic data and provided specific knowledge in the metabolomics field. All authors contributed to the article and approved the submitted version.
